# Detection and characterisation of visual field defects using Saccadic Vector Optokinetic Perimetry in children with brain tumours

**DOI:** 10.1038/s41433-018-0135-y

**Published:** 2018-06-07

**Authors:** Ian C. Murray, Conrad Schmoll, Antonios Perperidis, Harry M. Brash, Alice D. McTrusty, Lorraine A. Cameron, Alastair G. Wilkinson, Alan O. Mulvihill, Brian W. Fleck, Robert A. Minns

**Affiliations:** 1 University of Edinburgh, Edinburgh, UK; 2 Princess Alexandra Eye Pavilion, Edinburgh, UK; 3 Royal Hospital for Sick Children, Edinburgh, UK; 4 Heriot Watt University, Edinburgh, UK; 5 Glasgow Caledonian University, Glasgow, UK

## Abstract

**Purpose:**

To determine the ability of Saccadic Vector Optokinetic Perimetry (SVOP) to detect and characterise visual field defects in children with brain tumours using eye-tracking technology, as current techniques for assessment of visual fields in young children can be subjective and lack useful detail.

**Methods:**

Case-series study of children receiving treatment and follow-up for brain tumours at the Royal Hospital for Sick Children in Edinburgh from April 2008 to August 2013. Patients underwent SVOP testing and the results were compared with clinically expected visual field patterns determined by a consensus panel after review of clinical findings, neuroimaging, and where possible other forms of visual field assessment.

**Results:**

Sixteen patients participated in this study (mean age of 7.2 years; range 2.9–15 years; 7 male, 9 female). Twelve children (75%) successfully performed SVOP testing. SVOP had a sensitivity of 100% and a specificity of 50% (positive predictive value of 80% and negative predictive value of 100%). In the true positive and true negative SVOP results, the characteristics of the SVOP plots showed agreement with the expected visual field. Six patients were able to perform both SVOP and Goldmann perimetry, these demonstrated similar visual fields in every case.

**Conclusion:**

SVOP is a highly sensitive test that may prove to be extremely useful for assessing the visual field in young children with brain tumours, as it is able to characterise the central 30° of visual field in greater detail than previously possible with older techniques.

## Introduction

Central nervous system (CNS) tumours are the commonest solid tumours of childhood and the most common cause of childhood cancer death. Primary malignant brain tumours have an age-adjusted incidence rate of 3.27/100,000 in children aged 0–14 years at time of diagnosis. The most frequently occurring tumours are pilocytic astrocytomas (26%) and Primitive Neuroectodermal Tumours (PNET) including medulloblastoma (22%). Other gliomas (e.g. ependymoma, oligodendroglioma and choroid plexus tumours) occur less commonly [1]. Visual pathway gliomas account for 3–5% of all paediatric CNS tumours [2].

Current strategies for children with brain tumours aim to maintain survival rates with minimal treatment related long-term sequelae. Hence their management requires multidisciplinary team (MDT) input in treatment and monitoring [3]. These children commonly have visual field defects not reported by the child nor recognised by the care-giver [4]. When tumours involve the visual pathways, preservation of vision becomes a treatment objective. Careful ophthalmological follow-up is required in conjunction with other MDT specialties namely; paediatric neuro-oncology, neurology, neurosurgery, radiology and radiotherapy [5]. Ophthalmological assessment usually takes the form of fundoscopy including assessment of optic disc appearances, along with visual acuity (VA), colour vision and visual field testing. Objective visual field testing is valuable in demonstrating stability or progression of a tumour, particularly if the tumour involves the anterior visual pathway, and if visual field changes are corroborated by MRI appearances effective clinical management decisions can be made. Visual field information also allows practical advice to be provided to assist a child’s daily living.

Despite the potential benefits, there is a dearth of effective visual field testing methods appropriate for children under 5 years that can provide reliable detail greater than that gained from the confrontation method. Confrontation in children typically requires one examiner to gain a child’s fixation, and a second examiner to introduce dynamic stimuli into various part of the child’s visual field from the rear and side while the first examiner observes the child’s gaze for a reaction. The technique allows only an approximate assessment of visual field defects and in children its usefulness is limited by the attention and concentration of the child. Obtaining reliable and objective field testing in healthy young children is challenging and frequently more difficult in children who are ill as a result of brain tumours. The difficulties associated with performing standard automated perimetry (SAP) in children are well known. The main problems children have with SAP are understanding how to perform the test and maintaining central fixation [6, 7]. Manual kinetic perimetry is more popular in children between the ages of 5 and 9 years because the test can be tailored to the child’s ability [8, 9]. However, the technique still requires the child’s cooperation and understanding, and results can be dependent upon the examiner’s testing skills [10]. More recently, a modified form of arc perimetry, the behavioral visual field (BEFIE) test, has been assessed in a large cohort of children [11]. BEFIE was found to be a useful tool for detecting visual field defects in young or neurologically impaired children. However, the technique is dependent on the skills of an examiner and an observer, and is not suited to identifying absolute scotomas or relative defects.

Saccadic Vector Optokinetic Perimetry (SVOP) is an objective visual field assessment technique developed specifically for young children unable to perform conventional forms of perimetry [12, 13]. It uses a multi-fixation target strategy, in combination with modern eye-tracking technology to monitor real-time fixations and eye movement responses to visual field stimuli. SVOP makes automated decisions on whether or not visual field stimuli have been seen based on a child’s natural eye movements. The child does not need to maintain fixation on a single target, nor do they need to make responses with a push button. Hence, there is no great requirement for understanding the test. SVOP continuously monitors patient position and can present visual stimuli at specific visual field locations even if the patient moves during the test meaning that the child’s head does not need to be placed on a chinrest.

The aim of this study was to determine the feasibility of SVOP in the detection and characterisation of visual field defects in children with brain tumours, and to authenticate the SVOP results by comparing them with clinically expected visual field patterns following review of neuroimaging, and where possible other forms of visual field assessment.

## Methods

### Patients

The families of children with known or suspected brain tumours managed at the Royal Hospital for Sick Children in Edinburgh from April 2008 to August 2013 were invited to participate in the study.

### Study method

Each patient underwent binocular SVOP visual field testing. In addition, monocular testing was also performed if the patient was considered of a suitable developmental ability. Many of the participants were tested repeatedly over months or years of follow-up. This study reports the first SVOP testing episode for each child.

All patients had undergone MRI neuroimaging as part of their oncological workup and ongoing treatment. A consensus panel comprising an experienced ophthalmologist (BF), neurologist (RM) and neuro-radiologist (GW), reviewed the MR images for the scanning session for each patient that was closest in time to their SVOP test date. Care was taken to ensure that none of the patients had undergone interventions such as radiotherapy or surgery in the interval between the MR imaging session and the SVOP test. The panel was masked to the SVOP results. Based on radiological appearances, and with clinical information including full clinical history, VA, ocular motility, optic disc appearances and results of other VF testing methods, e.g. confrontation or Goldmann (when available), the panel predicted an expected visual field deficit for each patient.

Additionally, an experienced ophthalmologist (CS), masked to patient details and imaging, independently interpreted and described the measured SVOP visual field for each child in the study. Abnormal visual fields were those which had two or more contiguous points “unseen” in the same quadrant or vertical hemifield, or three or more non-contiguous “unseen” points in one quadrant or vertical hemifield. If a visual field was classified as abnormal, the defect was categorised using standard neuro-ophthalmology visual field abnormality terms.

The panel-predicted visual fields were then compared with the SVOP results and a two-by-two contingency table was utilised to determine the sensitivity and specificity (and the positive and negative predictive values) of the SVOP test in detecting a clinically significant visual field abnormality.

When children became capable of performing Goldman visual field testing during follow-up, the pattern of visual field abnormality obtained using Goldman testing was compared with the pattern of visual field abnormality obtained using SVOP. The pair of tests with the shortest time interval between them was used.

### The SVOP technique

**Fig. 1:**
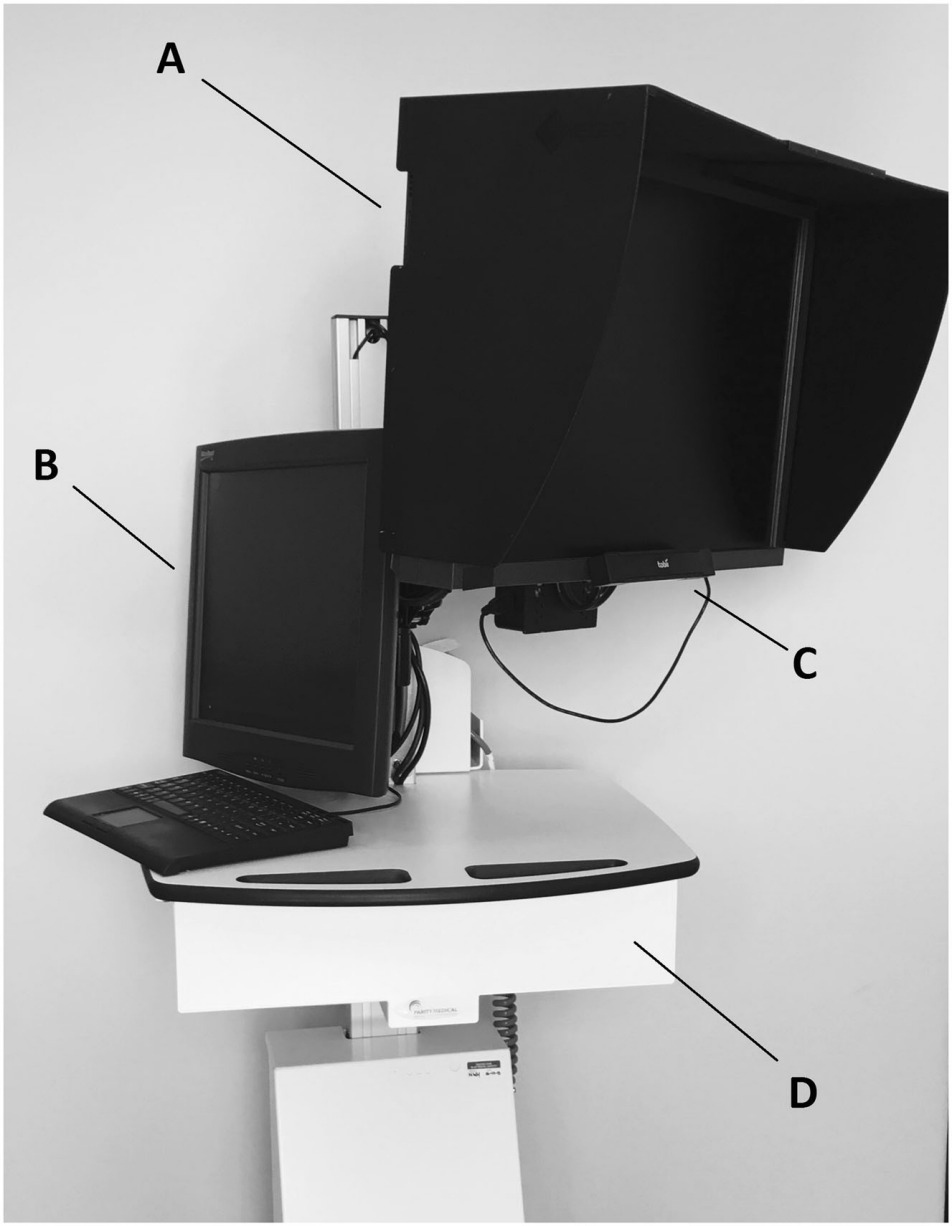
The Saccadic Vector Optokinetic Perimetry (SVOP) system components. **a** Patient display. **b** Examiner display. **c** Eye tracker. **d** Height adjustable surface housing the personal computer

The SVOP system comprises a personal computer (PC), a patient display (20” Liquid Crystal Display, Dell 2005FPW) and an eye tracker. During the course of this study two different models of eye tracker (X50 and IS-1 models from Tobii Technology) were used on different occasions. Figure 1 demonstrates the hardware.

Prior to any SVOP test, a calibration procedure was performed, to produce accurate gaze data for the patient being tested. During a calibration procedure, patients were required to look at the SVOP display screen and follow a visual stimulus with their gaze to five different screen locations. During this procedure, the eye tracker measures characteristics of the subject’s eyes (such as pupil position and shape) and used them together with a mathematical 3D eye model to calculate gaze position. The calibration stimulus was a cartoon character which moved to each of the five screen locations whereupon a simple short animation was played to hold the attention of the child. The calibration procedure lasted ~20–30 s.

**Fig. 2:**
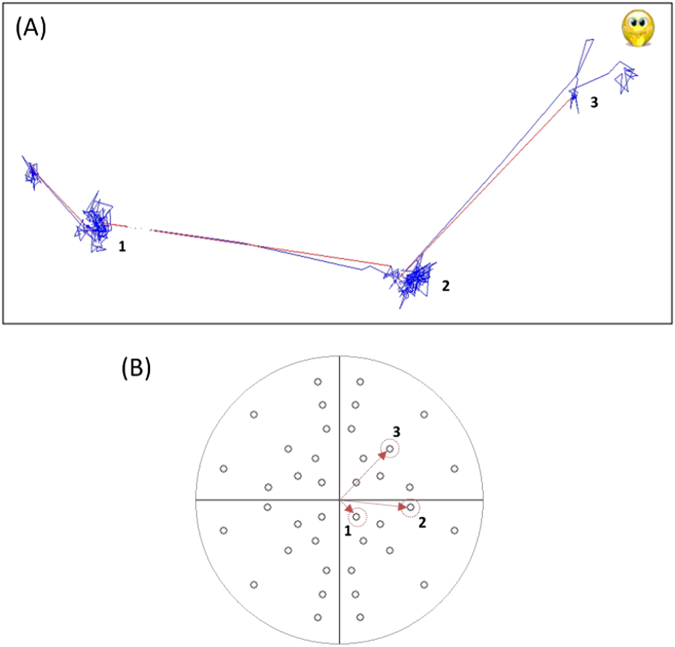
Example of eye gaze movements for three different visual field points which were all “seen” and a normal visual field plot. **a** Blue lines represent eye gaze movements made every 20 ms. Red lines represent a change in fixation (saccade) detected by SVOP. **b** A normal visual field plot (with all points “seen”). The three numbered points (highlighted with red arrows and circles) correspond to the fixation changes numbered in **a**

Each SVOP test began with a central fixation target. For older children a high contrast circular fixation target was used while for younger children the fixation target was a cartoon face to encourage attention and maintain interest. Fixation targets were of an angular diameter of ~1.5°. Real-time gaze data allowed the system to identify when the patient was looking at the fixation target. The instant this was detected, the fixation target disappeared and a test stimulus was presented at a location in their visual field. The on-screen location and size of the test stimulus was calculated in real-time and was based on the visual field angle to be tested and the position of the patient’s eyes relative to the fixation target at that instant. A software algorithm (details previously published [13]) analysed the direction and amplitude of any subsequent saccades completed within a time window of 1 s. If an analysed saccade related to where the stimulus was shown, this was recorded as a “seen” stimulus and a new fixation target was presented at the location of the test stimulus and the process was be repeated to assess another visual field location. Figure 2 shows an example of three “seen” visual field points and the associated gaze data used during an SVOP test.

If no gaze response, or an inaccurate saccade, occurred following the presentation of a visual field stimulus, the point was retested at a later stage in the test. Upon retesting, if the stimulus was not “seen” it was recorded as “unseen”. The visual field test stimuli were all of size Goldmann III (0.43° angular diameter) and duration 200 ms. Through the use of a calibrated LCD [14], the luminance of the test stimuli used and the background was 137 and 10 cd/m^2^, respectively. For reference, this stimulus brightness level is equivalent to 14 dB on the scale used by the Humphrey Field Analyser (HFA). The binocular test pattern used was a pattern consisting of 40 points (with 10 points in each quadrant) arranged with eccentricity out to 25° (Fig. 2b). The monocular test patterns used were equivalent to the 40-point (C40) screening test pattern used on the HFA, with the addition of an extra test point located at the blind spot.

This study was approved by the Lothian Regional Ethics Committee. Signed consent was obtained from all participants and/or their parent or guardian.

## Results

Sixteen (seven male, nine female) patients already receiving treatment and follow-up for brain tumours participated in this study. The mean age at the time of the reported SVOP test was 7.2 years (range 2.9–15 years). Patients 4, 8 and 9 were tested using an IS-1 eye tracker while the remaining thirteen patients were tested using an X50 eye tracker. The average test time for completed tests was 5 min 4 s (range 1 min 5 s to 9 min 7 s). The large range in test times can be attributed to the different testing strategies used for younger and older children and also the size of visual field defect present. Younger children were presented with small cartoon animations when they successfully detected visual field test stimuli. This animation sequence introduced an additional 2–3 s per “seen” stimulus. In addition, “unseen” stimuli are always retested. Consequently, a patient with a complete hemianopia would have half their visual field points retested, thus lengthening the test time.

The average interval between the SVOP test and the closest neuroimaging session, used by the consensus panel, was 52 days (range 5–146 days).

Twelve patients (detailed in Tables 1 and 2) out of sixteen (75%) successfully performed the SVOP test. SVOP testing failed due to poor quality eye tracking in four patients. In two of these patients the eye tracker was unable to detect the eyes sufficiently to perform SVOP testing, while in the other two patients the eyes were detected but the gaze data showed an offset when compared with the coordinates of the on-screen fixation targets.

**Table 1:** The ten patient cases where panel-predicted visual field were consistent with SVOP results

	Patient demographics		Ophthalmology assessment outcomes			Neuroimaging outcomes		SVOP outcomes
Case	Diagnosis and procedures prior to SVOP test	Age at SVOP test (years)	VA Right	VA Left	Confrontation and/or Goldmann visual field	Imaging description	Panel-predicted visual field on binocular testing	SVOP description
1	1. Left optic nerve/hypothalamic pilocytic astrocytoma	5.5	6/9	NPL	**Confrontation** (April 2008) Complete temporal hemianopia of right eye	(July 2008)	Right hemianopia, may have some residual right-sided function	Binocular (May 2008) Right superior quadrantanopia
	2. Biopsy at diagnosis					T1 post gadolinium axial image showed suprasellar hypothalamic enhancing lesion adjacent to left chiasm		
	3. Chemotherapy completed October 2006							
2	1. Right optic nerve/hypothalamic pilocytic astrocytoma	2.9	PL	6/9	**Confrontation** Left eye complete temporal defect. Right eye “impossible” to test (October 2008)	(August 2008)	Left hemianopia, could have subtle right visual field loss in addition	Binocular (October 2008) Left temporal hemianopia with missed points right inferior quadrant
	2. Right frontal craniotomy with subtotal removal January 2008					T1 post gadolinium axial image showed residual postoperative suprasellar cystic lesion with enhancing soft tissue abutting right chiasm and right internal carotid artery		
4	1. NF1	3.8	6/9	6/9	No information available	(July 2012)	Normal field, could have patchy loss	Binocular (August 2012) Inferior scattered loss
	2. Spectacles for accommodative esotropia					Coronal FLAIR image showed asymmetric thickening of optic nerves and chiasm with extension into left thalamus		
	3. Optic chiasm glioma							
5	1. Right parieto-occipital high grade glioma	5.1	6/6	6/6	**Confrontation** fields full. (October 2011)	(January 2012)	Left hemianopia, could have superior sparing	Binocular (December 2011) Left inferior quadrantanopia
	2. Surgical resection September 2011				**Goldmann** unable to perform (January 2012)	T2 axial image showed Surgical resection cavity in right parieto-occipital lobe		
	3. Focal cranial radiotherapy completed November 2011							
7	1. Hypothalamic ependymoma	3.2	6/6	NPL	**Confrontation** Difficult to test visual function (February 2010)	(September 2009)	Right hemianopia, could have some left sided loss	Binocular (November 2009)
	2. Fronto-temporal craniotomy and debulking of left suprasellar mass July 2009					T1 post gadolinium axial image showed prominent right optic nerve with residual tumour in suprasellar cistern		Right hemianopia and random scattered left hemifield missed points
14	1. Left fronto-temporal anaplastic ependymoma	6.3	6/5	6/9	**Confrontation** Examination normal (May 2011)	(April 2011)	Right hemianopia, may have inferior sparing	Left eye (March 2011) Scattered superior and nasal loss on left monocular visual field test. Right monocular and binocular visual field both normal
	2. Craniotomy and excision of tumour December 2009; subsequent repeat craniotomy and excision of recurrence March 2010					T1 post gadolinium axial image showed evidence of previous surgery and radiotherapy in left temporal lobe		
	3. Cranial radiotherapy							
15	1. Hypothalamic pilocytic astrocytoma	15	6/36	6/5	**Goldmann** showed incomplete left homonymous hemianopia. Some residual vision to left of vertical midline (June 2011)	(January 2011)	Left hemianopia	Left eye (February 2011) Left hemianopia
	2. Biopsy and right ventriculoperitoneal (VP) shunt April 2010; Left VP shunt August 2010					T1 post gadolinium axial image showed hypothalamic tumour with central necrosis and peripheral enhancement post-radiotherapy		
	3. Focal radiotherapy November 2010							
16	1. Left temporal pilocytic astrocytoma	4.4	6/5	6/6	**Confrontation** showed signs of right homonymous hemianopia	(September 2009)	Right hemianopia	Binocular (October 2009) Right hemianopia with missed points on left side
	2. Left fronto-temporal craniotomy and debulking September 2009				(October 2009)	T2 axial image showed left temporal resection cavity with medial extension of residual tumour in left thalamus and compression of chiasm		
3	1. NF1	10.7	6/6	6/6	**Goldmann** Within normal limits (April 2012)	(September 2011)	Normal field	Binocular (February 2012) Normal field
	2. Right optic tract thickening—possibly small glioma, T2 hyperintensity Left internal capsule					Coronal FLAIR image showed T2 hyperintensities in the globus pallidus bilaterally, in keeping with NF1		
	3. Poor motor co-ordination and dyspraxia							
6	1. Posterior fossa ependymoma - mainly L cerebellar pontine angle	5.2	6/6	6/6	**Confrontation** fields full (June 2010)	(January 2011)	Normal field	Binocular (January 2011) Normal field
	2. Posterior fossa craniotomy and complete excision November 2009					T1 post gadolinium axial image showed left posterior fossa surgical resection cavity		
	3. Proton beam radiotherapy completed March 2010							

Patients with abnormal visual fields are listed before those with normal fields

Details included are (1) Patient demographics (diagnosis and procedures prior to SVOP test and age at SVOP test), (2) Ophthalmology assessment outcomes (VA and confrontation and Goldmann perimetry if attempted), (3) Neuroimaging outcomes (a scan image and subsequent panel-predicted visual field), and (4) The SVOP test outcomes (SVOP plot and visual field description).

*VA* visual acuity, *NPL* no perception of light, *PL* perception of light, *NF1* Neurofibromatosis type 1, *SVOP* Saccadic Vector Optokinetic Perimetry

**Table 2:** The two patient cases where panel-predicted visual field were not consistent with SVOP results

	Patient demographics		Ophthalmology assessment outcomes			Neuroimaging outcomes		SVOP outcomes
Case	Diagnosis and procedures prior to SVOP test	Age at SVOP test (years)	VA Right	VA Left	Confrontation and/or Goldmann visual field	Imaging description	Panel-predicted visual field on binocular testing	SVOP description
8	1. Watson syndrome (combined Noonan syndrome and NF1)	5.7 y	6/6	NPL	**Confrontation** Good peripheral field of vision (August 2012)	(August 2012)	Normal field	Right eye (June 2012) Random scattered missed points
	2. Complete excision of left optic nerve pilocytic astrocytoma February 2010					T2 axial image showed high signal in the right brainstem (arrow) and bilateral subtle high signal in the medial temporal lobes in the regions of the optic tracts		
	3. Healthy right optic nerve head							
9	1. Medulloblastoma; midline cerebellum	7.6 y	6/6	6/6	No information available	(May 2013) Coronal FLAIR image showed Postsurgical atrophy of right cerebellar hemisphere with prominent 4th ventricle	Normal field	Binocular (July 2013) Constricted
	2. Complete excision June 2008							
	3. Right 6th nerve palsy							
	4. Chemotherapy completed January 2009							

Details included are (1) Patient demographics (diagnosis and procedures prior to SVOP test and age at SVOP test), (2) Ophthalmology assessment outcomes (VA and confrontation and Goldmann perimetry if attempted), (3) Neuroimaging outcomes (a scan image, description and subsequent panel-predicted visual field), and (4) The SVOP test outcomes (SVOP plot and visual field description).

*VA* visual acuity, *NPL* no perception of light, *NF1* Neurofibromatosis type 1, *SVOP* Saccadic Vector Optokinetic Perimetry

In the cases where the eye tracker could not detect the eyes sufficiently, one patient was wearing heavy mascara and the implications of this was realised subsequent to testing. The use of mascara is known to be a problem for eye tracking because it can prevent the accurate detection of the pupils [15]. This patient was not available for further testing due to geographical constraints. The second patient had a diagnosis of neurofibromatosis-1 and had congenital glaucoma with buphthalmos causing a cloudy cornea in one eye. The eye tracker was therefore prevented from tracking the eye due to the opaque nature of the cornea.

In the two patients where poor quality eye tracking was due to an offset in the gaze data rather than poor detection of the eyes, one patient had extremely poor VA and unsteady fixation secondary to marked optic atrophy. The final patient with a failed test had a right hemiplegia with poor co-ordination and would be expected to have a corresponding right hemianopia. However, a reason for the observed disparity between gaze coordinates and displayed fixation targets has not been elucidated in this patient.

In each of these four patients, the SVOP system was not able to determine that the patient was correctly fixating on the displayed fixation targets (due either to lack of gaze data or gaze data that did not correspond to the fixation target). The decision to abort these tests was made by the tester when it became clear that the test was not proceeding correctly. This was known because the system would continually try to regain the child’s fixation and not test any visual field points.

A two-by-two contingency table was used to compare the gross correlation of ‘normal’ versus ‘abnormal’ visual field between the consensus panel prediction and SVOP findings for the twelve successfully completed tests. (Table 3) There were eight true positives, two true negatives, and two false positive results. There were no false negatives. In this series, SVOP therefore had sensitivity for detecting an abnormal visual field of 100% and a specificity of 50% (positive predictive value of 80% and negative predictive value of 100%).

**Table 3:** The panel-predicted visual field outcome compared with the SVOP outcome for the twelve successfully performed tests

	Visual field prediction from imaging (panel decision)	
	Abnormal visual field	Normal visual field
SVOP result		
Abnormal visual field	8	2
Normal visual field	0	2

Table 1 details the ten cases where panel-predicted visual field were consistent with SVOP results (the eight true positives are detailed prior to the two true negatives). The remaining two patients had SVOP results inconsistent with the panel-predicted visual field. Table 2 details these two false positive results. For further information, including corresponding neuroimaging and SVOP plots, please see the online supplementary.doc versions of Tables 1 and 2, at *Eye* journal’s website.

Patients 1, 2, 7, 8 and 15 were effectively monocular due to poor or absent VA in one eye as a result of optic nerve or chiasmal involvement by tumour. Patients 3, 5, 9 and 14 all completed monocular and binocular SVOP tests; the test which best demonstrates the VF abnormality has been included in the table. Only three of the younger patients (4, 6 and 16) who were unable to comply with monocular occlusion, had purely binocular SVOP testing.

**Fig. 3:**
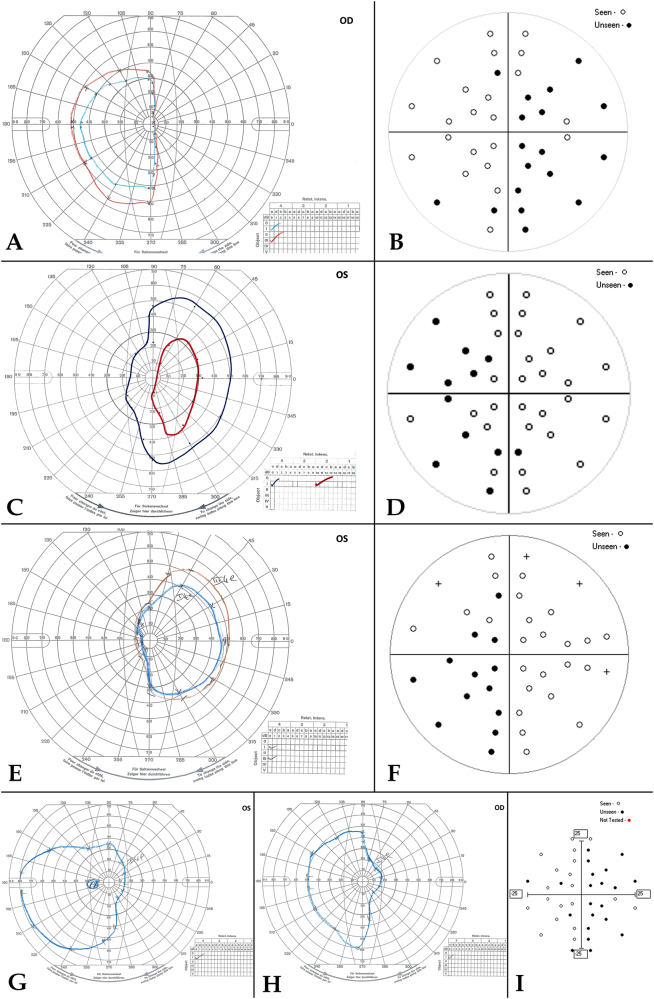
Children with abnormal visual fields, who became capable of Goldman perimetry during follow-up. A comparison of Goldmann and SVOP visual fields. **a**, **b** Patient 1. Left optic nerve/hypothalamic pilocytic astrocytoma with blind left eye. Goldman (**a**) and SVOP (**b**) confirm right temporal hemianopia in only seeing eye with residual right nasal field. On the SVOP plot, (○: seen, ●: unseen) **c**, **d** Patient 2. Right optic nerve/hypothalamic pilocytic astrocytoma with blind right eye. Goldman (**c**) and SVOP (**d**) confirm left temporal hemianopia in only seeing eye with residual left nasal visual field. **e**, **f** Patient 15. Hypothalamic pilocytic astrocytoma with blind right eye. Goldman (**e**) and SVOP (**f**) confirm left temporal hemianopia in seeing left eye, with residual left nasal field of vision. **g**–**i** Patient 16. Left temporal pilocytic astrocytoma. Goldmann (**g, h**) and binocular SVOP (**i**) confirm right-sided hemianopia

Six children (1, 2, 3, 14, 15 and 16) became capable of performing Goldmann Visual Field testing during follow-up. One had a right hemianopia on Goldmann testing, two patients had a left hemianopia, one had a right homonomous hemianopia, and two had a full visual field. The pattern of visual field abnormality present on SVOP testing was the same in every case. Figure 3 compares the Goldmann field results with the SVOP test for the four children with abnormal fields confirmed by Goldmann perimetry.

## Discussion

SVOP was successfully performed in the majority (75%) of this cohort of children with brain tumours, even in those under three years of age. Successful completion of a test was associated with 100% sensitivity in detecting a visual field defect when compared with ophthalmic assessment and/or panel consensus following review of neuroimaging. In most of these cases the specific character of the measured visual field defect was compatible with the expected visual field defect when considering the anatomy of the visual pathways. In case 14, the initial field defect detected by the first SVOP differed from the expected defect, despite a repeatable test. However, a nasal defect detected in that eye is plausible given that the gliotic scarring in the left temporal lobe is immediately adjacent to the temporal aspect of the prechiasmal left optic nerve. In this case the panel prediction was based on a predominantly retrochiasmal tumour when in fact the area of contact was in the anterior visual pathway.

Subsequent Goldmann and SVOP testing demonstrated recovery of a full visual field in either eye.

Overall, six children became capable of performing Goldmann visual fields testing during follow-up. In each case, the pattern of visual field abnormality was the same with Goldman testing and SVOP testing. This finding supports our contention that SVOP assessment at an early age in our cohort was reliable. We have previously published comparison studies of Humphrey Standard Automated Perimetry and SVOP in adults, with favourable results [16].

One of the false positive SVOP tests (case 8) showed poor quality eye tracking with a prolonged test time (9 min 7 s). A potential loss of interest and concentration from the child could have resulted in false positive algorithm decisions and scattered unseen points. However, the bilateral optic tract dysmyelination seen in this patient could have a measurable effect on the visual field. The constricted visual field defect recorded for the remaining false positive case (case 9), whilst not anticipated by the consensus panel, could in fact represent a true constriction due to previous raised intracranial pressure.

Test failure occurred in four children and the reasons for this have been elucidated. All four of the failures related to poor eye tracking. In two patients the eye tracker was unable to detect the eyes sufficiently, while the remaining two patient’s eyes were detected but the gaze data showed an offset when compared with the coordinates of the on-screen fixation targets. In each scenario, the SVOP system was not able to determine that the patient was correctly fixating on the displayed fixation targets and hence would not proceed to test visual field points. The decision to abort these tests was made by the tester when it became clear that the test was not proceeding in a satisfactory manner.

In patients where eye tracking is poor a complete result is often not possible. Moreover, it is possible that incomplete test results should be considered unreliable because of prolonged test time and reduced interest from the child. In future SVOP studies it will be important to assess the factors that contribute to unreliable tests so that a useful indication can be provided to the tester.

In this study, three patients were tested using a different model eye tracker than the remaining patients. Improvements to eye-tracking technology are continuingly being made and it was thought that a newer model of eye tracker would improve the SVOP test. Both of the eye trackers used in this study have since been discontinued by Tobii Technology due to continuing development of their eye trackers and our research group have more recently been performing studies using another eye tracker model (X2-60 also from Tobii Technology).

There are few reliable alternatives for assessing visual field loss in young children which can show the same level of detail as SVOP. Manual visual field testing approaches, such as that used by the BEFIE test [11] or the KidzEyes preferential looking technique [17], have the advantage that they can obtain visual field information for children who cannot be adequately eye tracked with SVOP. However, they are unable to accurately test specific visual field locations. The use of Visual Evoked Potentials (VEPs) for the detection and monitoring of visual pathway tumours in children has not shown consistent results [18, 19]. Avery and colleagues have demonstrated that vision loss in children with visual pathway gliomas is associated with measurable defects of the retinal nerve fibre layer (RNFL), as determined by optical coherence tomography (OCT) [20, 21].

An advantage of SVOP is its ability to characterise the nature and extent of visual field defects, which have important functional implications for the child and it may be particularly useful if changes in tumour size or characteristics seen in neuroimaging are accompanied by functional visual field change detected by SVOP. The automated nature of SVOP means that minimal experience is required to perform the test or operate the system. Children find the test easy, and as it takes ~5 min to perform. With the use of engaging animations to hold and maintain concentration, a meaningful test result was obtained in the majority of cases. In this small cohort SVOP testing has demonstrated the potential for monitoring visual field changes in young patients with brain tumours in greater detail than was previously possible.

Future studies using SVOP will focus on longitudinal follow-up of a cohort of children with visual pathway tumours to (i) determine the repeatability and reliability of the test, and (ii) demonstrate changes of visual field defects in relation to progression of tumour size over time, and response to medical or surgical interventions. For anterior visual pathway tumours, the use of OCT may be used to confirm focal deficits in the RNFL corresponding to visual field defects mapped by SVOP.

### Summary

#### What was known before

- Assessment of visual fields is an important component of the assessment of visual pathway tumours in peadiatric neuro-oncology.
- Previous commonly used techniques such as confrontational visual field assessment lacked objective detail in young children, whilst standardised kinetic perimetry (Goldmann), or automated perimetry, were difficult for young children to perform reliably.
- Saccadic Vector Optokinetic Perimetry (SVOP) is a new, objective technique for visual field testing designed for young children.

#### What this study adds

- We assessed the ability of SVOP to detect expected or known visual field defects in a cohort of sixteen children with visual pathway tumours.
- SVOP demonstrated 100% sensitivity and 50% specificity when compared with expected visual field abnormality.
- The diagnosis and management of young children with visual pathway tumours would be aided by SVOP testing due to its ability to characterise the central visual field in detail.

## Electronic supplementary material

Supplementary Files
