# Supplementary material for: Detection and characterisation of visual field defects using Saccadic Vector Optokinetic Perimetry in children with brain tumours
Source: Eye (Lond). 2018 Jun 7;32(10):1563–73. doi: 10.1038/s41433-018-0135-y (PMC6169726; doi:10.1038/s41433-018-0135-y)
Supplement: Supplementary file 1 — Supplementary Files [file 41433_2018_135_MOESM1_ESM.docx]

SUPPLEMENTARY PUBLICATION VERSIONS

| **Table 1.** The ten patient cases where panel predicted visual field were consistent with SVOP results. Patients with abnormal visual fields are listed before those with normal visual fields. Details included are (1) Patient demographics (diagnosis and procedures prior to SVOP test and age at SVOP test), (2) Ophthalmology assessment outcomes (visual acuity and confrontation and Goldmann perimetry if attempted), (3) Neuroimaging outcomes (a scan image and subsequent panel predicted visual field), and (4) The SVOP test outcomes (SVOP plot and visual field description).  Abbreviations: VA (visual acuity), NPL (no perception of light), PL (perception of light), NF1 (Neurofibromatosis type 1), SVOP (Saccadic Vector Optokinetic Perimetry). | | | | | | | | | |
| --- | --- | --- | --- | --- | --- | --- | --- | --- | --- |
|  | **Patient demographics** | | **Ophthalmology assessment outcomes** | | | **Neuro-imaging outcomes** | | **SVOP outcomes** | |
| **Case** | **Diagnosis and procedures prior to SVOP test** | **Age at SVOP test (years)** | **VA Right** | **VA Left** | **Confrontation and/or Goldmann visual field** | **Imaging description** | **Panel predicted visual field on binocular testing** | **SVOP plot**  (○ : Seen, ●: Unseen,  + : Untested) | **SVOP description** |
| 1. | 1. Left optic nerve/hypothalamic pilocytic astrocytoma  2. Biopsy at diagnosis  3. Chemotherapy completed October 2006 | 5.5 | 6/9 | NPL | **Confrontation**  (April 2008)  Complete temporal hemianopia of right eye | 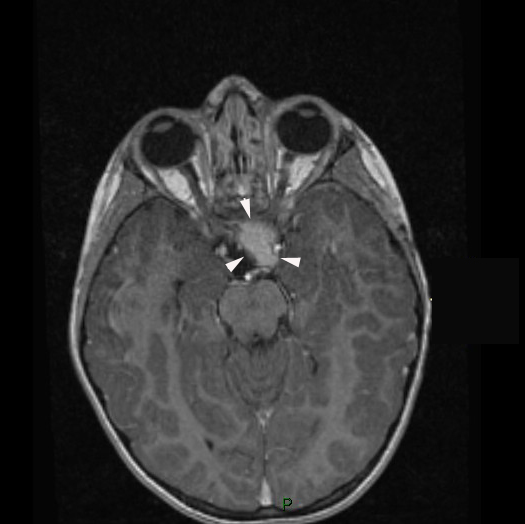  (July 2008)  T1 post gadolinium axial image showed suprasellar hypothalamic enhancing lesion adjacent to left chiasm | Right hemianopia, may have some residual right sided function | Binocular  (May 2008)  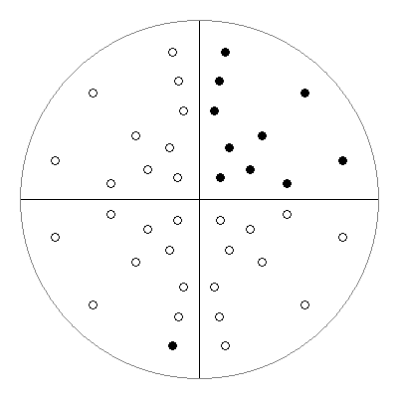 | Right superior quadrantanopia |
| **Table 1.** The ten patient cases where panel predicted visual field were consistent with SVOP results (continued). | | | | | | | | | |
| 2. | 1.Right optic nerve/hypothalamic pilocytic astrocytoma  2.Right frontal craniotomy with subtotal removal January 2008 | 2.9 | PL | 6/9 | **Confrontation**  Left eye complete temporal defect. Right eye “impossible” to test  (October 2008) | 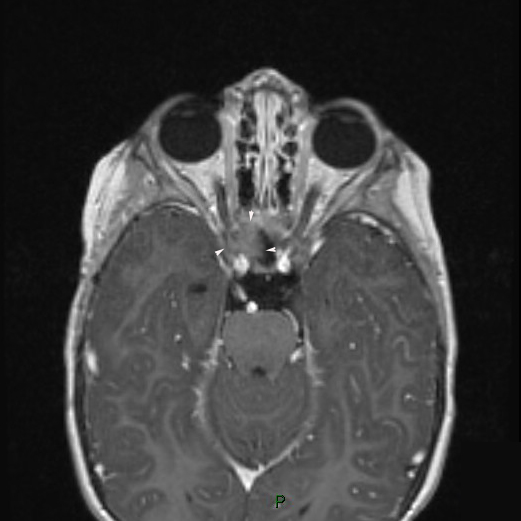  (August 2008)  T1 post gadolinium axial image showed residual postoperative suprasellar cystic lesion with enhancing soft tissue abutting right chiasm and right internal carotid artery | Left hemianopia, could have subtle right visual field loss in addition | Binocular  (October 2008)  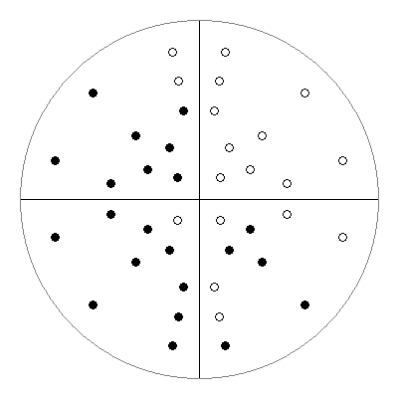 | Left temporal hemianopia with missed points right inferior quadrant |
| 4. | 1. NF1  2. Spectacles for accommodative esotropia  3. Optic chiasm glioma | 3.8 | 6/9 | 6/9 | No information available | 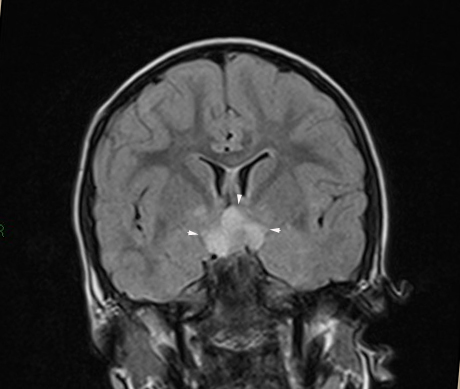  (July 2012)  Coronal FLAIR image showed asymmetric thickening of optic nerves and chiasm with extension into left thalamus | Normal field, could have patchy loss | Binocular  (August 2012)  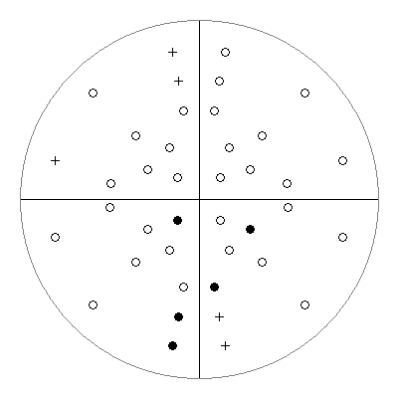 | Inferior scattered loss |
| 5. | 1. Right parieto-occipital high grade glioma  2. Surgical resection September 2011  3. Focal cranial radiotherapy completed November 2011 | 5.1 | 6/6 | 6/6 | **Confrontation** fields full.  (October 2011)  **Goldmann** unable to perform  (January 2012) | 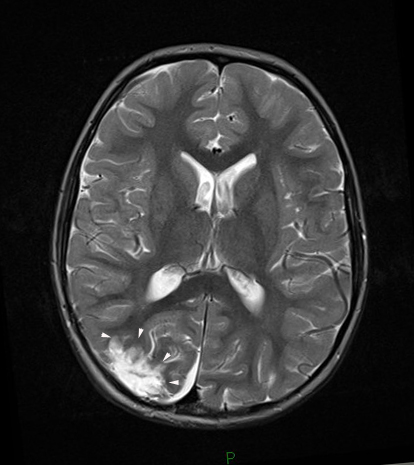  (January 2012)  T2 axial image showed Surgical resection cavity in right parieto-occipital lobe | Left hemianopia, could have superior sparing | Binocular  (December 2011)  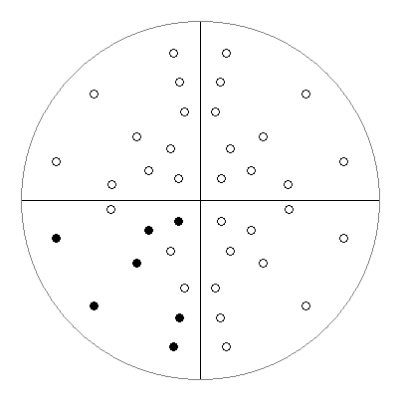 | Left inferior quadrantanopia |
| **Table 1.** The ten patient cases where panel predicted visual field were consistent with SVOP results (continued). | | | | | | | | | |
| 7. | 1. Hypothalamic ependymoma  2. Fronto-temporal craniotomy and debulking of left suprasellar mass July 2009 | 3.2 | 6/6 | NPL | **Confrontation** Difficult to test visual function  (February 2010) | 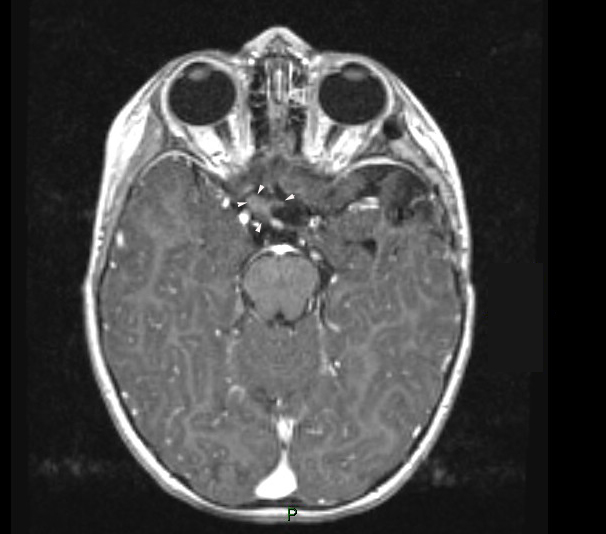  (September 2009)  T1 post gadolinium axial image showed prominent right optic nerve with residual tumour in suprasellar cistern | Right hemianopia, could have some left sided loss | Binocular  (November 2009)  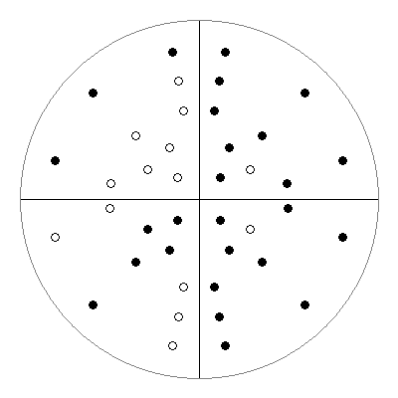 | Right hemianopia and random scattered left hemifield missed points. |
| 14. | 1. Left fronto-temporal anaplastic ependymoma  2.Craniotomy and excision of tumour December 2009; subsequent repeat craniotomy and excision of recurrence March 2010  3. Cranial radiotherapy | 6.3 | 6/5 | 6/9 | **Confrontation** Examination normal  (May 2011) | 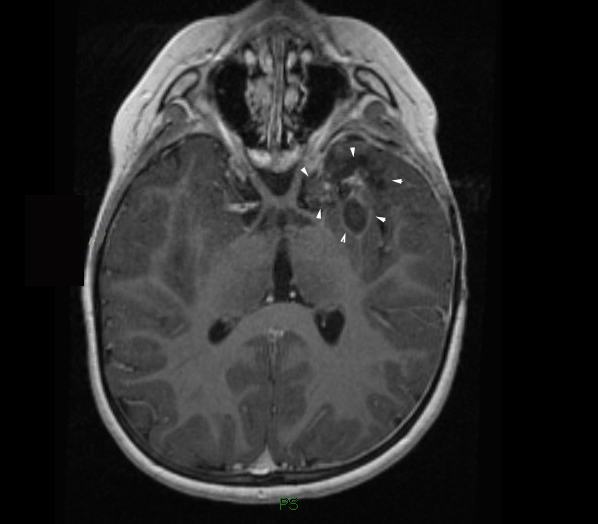  (April 2011)  T1 post gadolinium axial image showed evidence of previous surgery and radiotherapy in left temporal lobe | Right hemianopia, may have inferior sparing | Left eye  (March 2011)  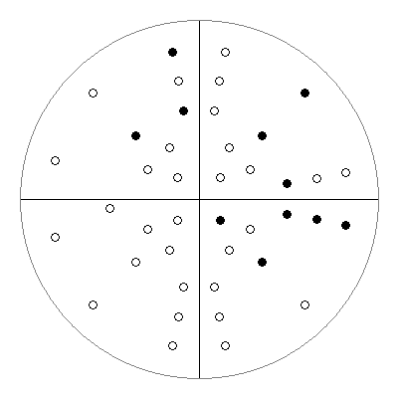 | Scattered superior and nasal loss on left monocular visual field test. Right monocular and binocular visual field both normal. |
| 15. | 1. Hypothalamic pilocytic astrocytoma  2. Biopsy and right ventriculoperitoneal (VP) shunt April 2010; Left VP shunt August 2010  3. Focal radiotherapy November 2010 | 15.0 | 3/36 | 6/5 | **Goldmann** showed incomplete left homonymous hemianopia. Some residual vision to left of vertical midline  (June 2011) | 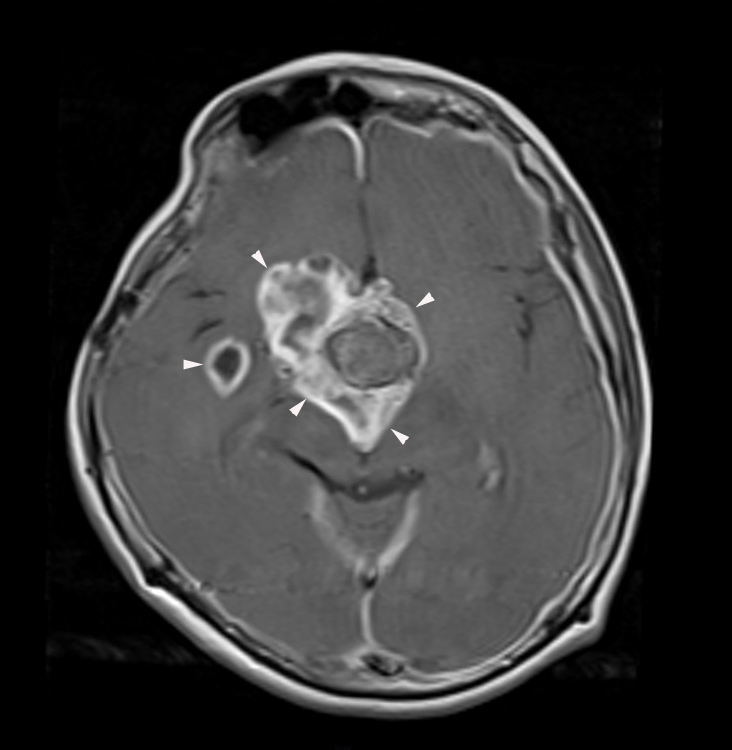  (January 2011)  T1 post gadolinium axial image showed hypothalamic tumour with central necrosis and peripheral enhancement post-radiotherapy | Left hemianopia | Left eye  (February 2011)  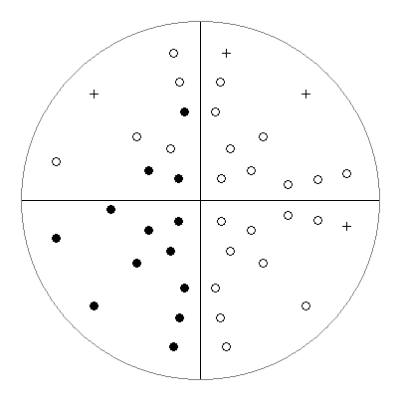 | Left hemianopia |
| **Table 1.** The ten patient cases where panel predicted visual field were consistent with SVOP results (continued). | | | | | | | | | |
| 16. | 1. Left temporal pilocytic astrocytoma  2. Left frontotemporal craniotomy and debulking September 2009 | 4.4 | 6/5 | 6/6 | **Confrontation** showed signs of right homonymous hemianopia  (October 2009) | 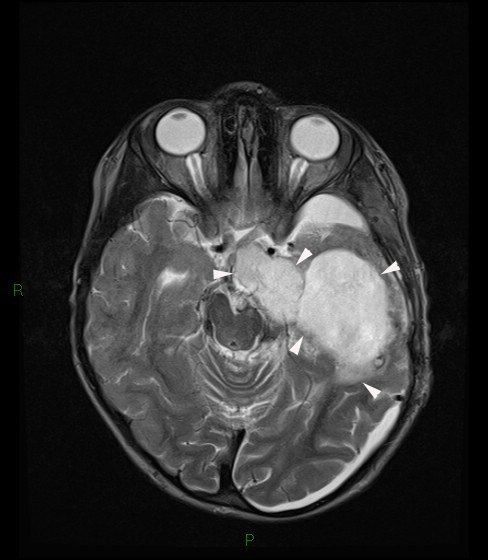  (September 2009)  T2 axial image showed left temporal resection cavity with medial extension of residual tumour in left thalamus and compression of chiasm | Right hemianopia | Binocular  (October 2009)  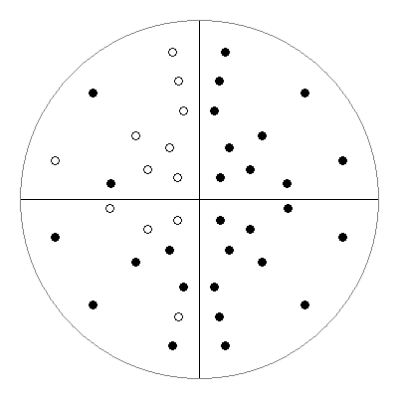 | Right hemianopia with missed points on left side |
| 3. | 1. NF1  2. Right optic tract thickening - possibly small glioma, T2 hyperintensity Left internal capsule  3. Poor motor co-ordination and dyspraxia | 10.7 | 6/6 | 6/6 | **Goldmann**  Within normal limits  (April 2012) | 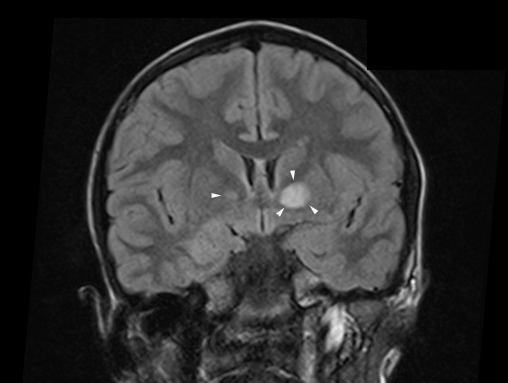  (September 2011)  Coronal FLAIR image showed T2 hyperintensities in the globus pallidus bilaterally, in keeping with NF1 | Normal field | Binocular  (February 2012)  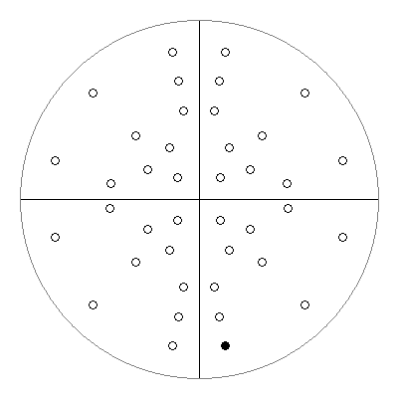 | Normal field |
| 6. | 1. Posterior fossa ependymoma - mainly L cerebellar pontine angle  2. Posterior fossa craniotomy and complete excision November 2009  3. Proton beam radiotherapy completed March 2010 | 5.2 | 6/6 | 6/6 | **Confrontation** fields full  (June 2010) | 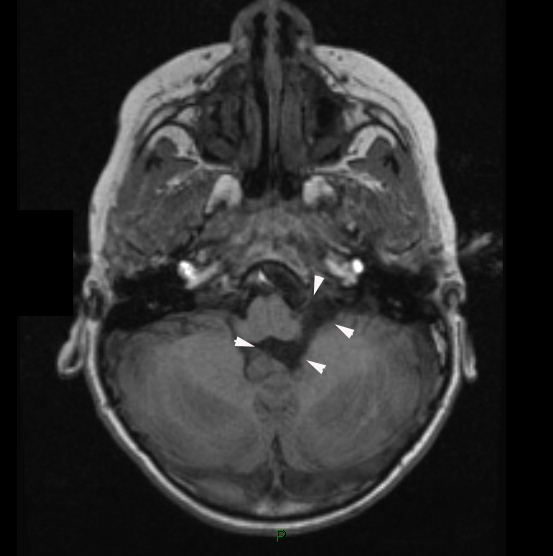  (January 2011)  T1 post gadolinium axial image showed left posterior fossa surgical resection cavity | Normal field | Binocular  (January 2011)  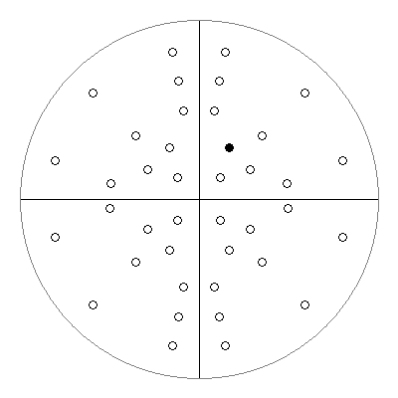 | Normal field |

| **Table 2.** The two patient cases where panel predicted visual field were not consistent with SVOP results. Details included are (1) Patient demographics (diagnosis and procedures prior to SVOP test and age at SVOP test), (2) Ophthalmology assessment outcomes (visual acuity and confrontation and Goldmann perimetry if attempted), (3) Neuroimaging outcomes (a scan image, description and subsequent panel predicted visual field), and (4) The SVOP test outcomes (SVOP plot and visual field description).  Abbreviations: VA (visual acuity), NPL (no perception of light), NF1 (Neurofibromatosis type 1), SVOP (Saccadic Vector Optokinetic Perimetry). | | | | | | | | | |
| --- | --- | --- | --- | --- | --- | --- | --- | --- | --- |
|  | **Patient demographics** | | **Ophthalmology assessment outcomes** | | | **Neuro-imaging outcomes** | | **SVOP outcomes** | |
| **Case** | **Diagnosis and procedures prior to SVOP test** | **Age at SVOP test (years)** | **VA Right** | **VA Left** | **Confrontation and/or Goldmann visual field** | **Imaging description** | **Panel predicted visual field on binocular testing** | **SVOP plot** | **SVOP description** |
| 8. | 1. Watson syndrome (combined Noonan syndrome and NF1)  2. Complete excision of left optic nerve pilocytic astrocytoma February 2010  3. Healthy right optic nerve head | 5.7 y | 6/6 | NPL | **Confrontation** Good peripheral field of vision  (August 2012) | 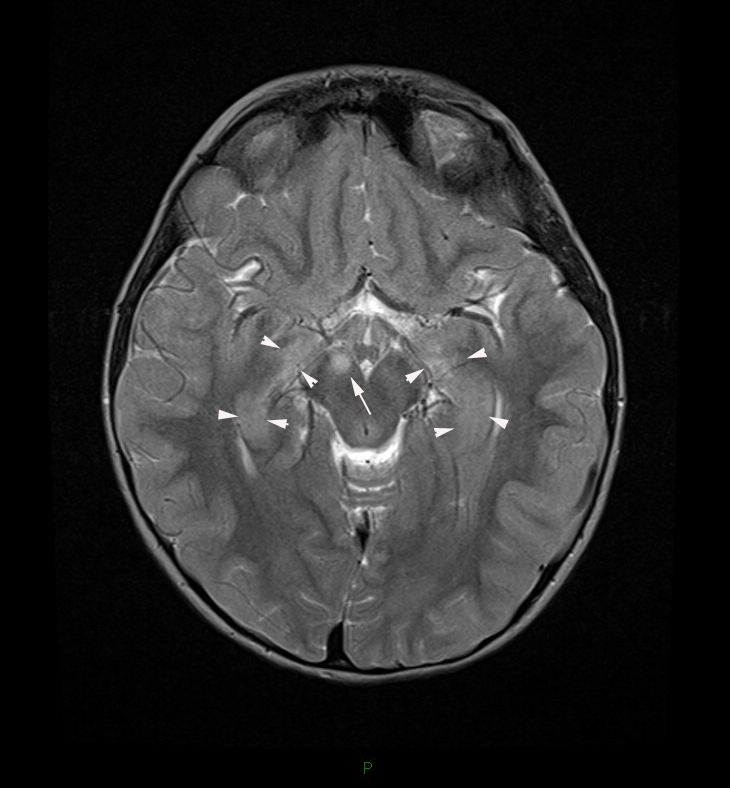  (August 2012)  T2 axial image showed high signal in the right brainstem (arrow) and bilateral subtle high signal in the medial temporal lobes in the regions of the optic tracts | Normal field | Right eye  (June 2012)  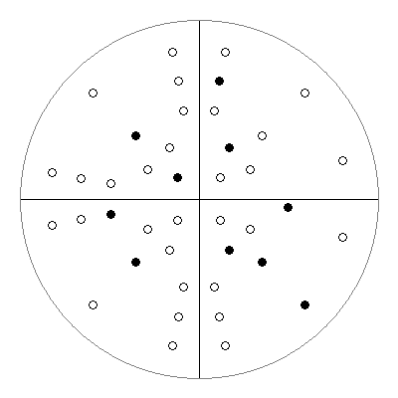 | Random scattered missed points |
| 9. | 1. Medulloblastoma; midline cerebellum  2. Complete excision June 2008  3. Right 6th nerve palsy  4. Chemotherapy completed January 2009 | 7.6 y | 6/6 | 6/6 | No information available | 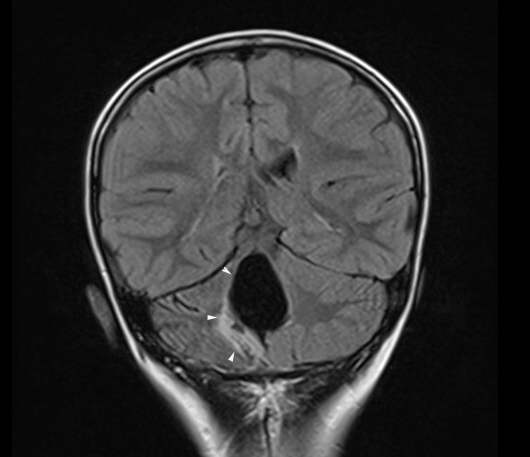  (May 2013)  Coronal FLAIR image showed Postsurgical atrophy of right cerebellar hemisphere with prominent 4^th^ ventricle | Normal field | Binocular  (July 2013)  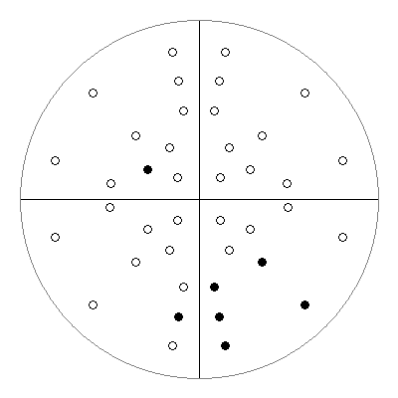 | Constricted |
